# P-1095. Impact of QI Infection Prevention and Control (IPC) Interventions on Hand Hygiene compliance and Clostridioides difficile infections (CDI)

**DOI:** 10.1093/ofid/ofaf695.1290

**Published:** 2026-01-11

**Authors:** Ashlesha Kaushik, Erin Mothershead, Kendra Goeden, Sandeep Gupta, Corey Thieman, Debra Blyth-Wilk

**Affiliations:** UnityPoint Health and University of Iowa Carver College of Medicine, Sioux City, IA; Unity Point Health, Sioux City, Iowa; Unity Point Health, Sioux City, Iowa; Pulmonary and Critical Care Medicine, Unity Point Health, Sioux City, Iowa; Unity Point Health at St. Luke's Regional Medical Center, Sioux City, Iowa; Spaulding Rehabilitation, MGB, Harvard Medical School, Boston, Massachusetts

## Abstract

**Background:**

Despite using CDC diagnostic criteria and Antimicrobial stewardship interventions we witnessed an increase in local CDI.

**Methods:**

Multifaceted IPC QI-interventions were implemented at our tertiary-care-center ICU Rehabilitation serving tristate-area of upper Midwest: manual-auditing, real-time observations for HCP hand-hygiene compliance; soap/sanitizer usage; education, hand-hygiene reminders in medical staff lounge, patient-rooms, nursing-stations, provider coaching and huddles focusing on nursing education. Pre-intervention-period (P1:4/1/2023- 9/30/2023) was compared with Intervention-period (P2:10/1/2023-3/31/2024). Additionally, *observation of automated hand hygiene system and a pilot Cross-sectional comparison was done for overall hand-hygiene compliance and *CDI* in our health-system (with manual auditing/monitoring) with a health-system with automated hand-hygiene monitoring [Spaulding Rehabilitation Center (Harvard Medical School, Boston, MA), using automated hand-hygiene system (*Biovigil^®^*) with inbuilt signaling/recording if improper hand-hygiene performed] (*supported by CDC PFL IPC Ambassador grant)

**Results:**

Hand-hygiene compliance rates for HCP increased from 69% [191/277 (compliant/observations)] during P1 to 91% (463/510) during P2 (P< 0.0001); with physicians increasing from 70% to 89% (p< 0.05); nurses 71% to 93% (p< 0.001); midlevel- providers 69% to 88% (p< 0.05); ancillary-staff 61% to 87% (p< 0.01)]. Hand-soap usage increased from 43% to 74% in P2 (p< 0.05). *C. difficile* SIR (Standardized Infection Ratio) decreased from 1.6 during P1 to 0.4 during P2 (p< 0.05). Comparatively, Spaulding Rehabilitation Center with automated hand-hygiene system had overall hand-hygiene compliance rates of 97% from 10/1/2023-3/31/24 (compared to 91% in our system) and *C. difficil*e SIR of 0 compared with 0.4 in our system (p< 0.0001).

**Conclusion:**

With interventions, we observed improved hand-hygiene compliance and decreased *CDI* at our institution, however, greater hand- hygiene compliance and lower SIR were noted with an automated system. Next steps would be to continue auditing/interventions and further evaluate applicability of automated-systems including cost-benefit analysis

**Disclosures:**

All Authors: No reported disclosures

